# Overexpression of SLC34A2 is an independent prognostic indicator in bladder cancer and its depletion suppresses tumor growth via decreasing c-Myc expression and transcriptional activity

**DOI:** 10.1038/cddis.2017.13

**Published:** 2017-02-02

**Authors:** Wen Ye, Cui Chen, Ying Gao, Zou-Shan Zheng, Yi Xu, Miao Yun, Hui-Wen Weng, Dan Xie, Sheng Ye, Jia-Xing Zhang

**Affiliations:** 1 Department of Oncology, The First Affiliated Hospital, Sun Yat-Sen University, Guangzhou, China; 2 The State Key Laboratory of Oncology in South China, Sun Yat-Sen University Cancer Center, Collaborative Innovation Center for Cancer Medicine, Guangzhou, China; 3 Department of Pathology, Sun Yat-Sen University Cancer Center, Guangzhou, China; 4 Department of Ultrasound, Sun Yat-Sen University Cancer Center, Guangzhou, China

## Abstract

Solute carrier family 34 member 2 (SLC34A2), a pH-sensitive sodium-dependent phosphate transporter, is associated with several human cancers. In this study, we investigate the clinical significance of SLC34A2 and its function in human bladder cancer (BC). The expression dynamics of SLC34A2 were examined in two independent cohorts of BC samples by quantitative PCR, western blotting and immunohistochemical staining. In the training cohort (156 cases), we applied the X-tile program software to assess the optimal cutoff points for biomarkers in order to accurately classify patients according to clinical outcome. In the validation cohort (130 cases), the cutoff score derived from X-title analysis was investigated to determine the association of SLC34A2 expression with survival outcome. A series of *in vitro* and *in vivo* assays were then performed to elucidate the function of SLC34A2 in BC and its underlying mechanisms. Results showed that SLC34A2 was significantly upregulated in BC cell lines and clinical samples. In both two cohorts of BC samples, high expression of SLC34A2 was associated with large tumor size, advanced T status and poor patients' survival. The depletion of SLC34A2 in BC suppressed cellular viability, colony formation and anchorage-independent growth *in vitro*, and inhibited xenograft tumor growth *in vivo*, whereas overexpression of SLC34A2 had the converse effect. Simultaneously, downregulation of SLC34A2 decreased the transcriptional activity and protein expression level of c-Myc in BC cells, whereas restoration of c-Myc expression could compromise the anti-proliferation effect of SLC34A2 depletion. Furthermore, miR-214 was proved as a negative regulator of *SLC34A2*. Our present study illustrated that SLC34A2 has an important role in promoting proliferation and tumorigenicity of BC, and may represent a novel therapeutic target for this disease.

Bladder cancer (BC) is one of the most common cancer worldwide, with >386 300 new cases and 150 200 deaths each year.^1^ In China, the incidence and mortality rates are increasing annually.^2^ BC is a highly heterogeneous malignancy and associated with high recurrence and mortality rates. At present, information based on the tumor node metastases (TNM) stage classification and conventional pathological features are insufficient to evaluate disease outcome. Thus, a large amount of investigations on BC have been focused on the identification of promising molecular biomarkers that could precisely evaluate patients' progression risk and help clinicians to optimize individual therapeutic strategy.^3^ For instance, we previously demonstrated that high expression of Rab25 is associated with lymph node metastasis and inferior clinical outcome of BC patients.^4^ To date, however, the search for specific biomarkers in BC cells that have clinical/prognostic value is still substantially limited.

Solute carrier family 34 member 2 (SLC34A2) is a pH-sensitive sodium-dependent phosphate transporter. Studies have shown that it is highly expressed in small intestine, kidney and the type II alveolar epithelium cells (AT-II) of lung, where it takes part in the synthesis of AT-II pulmonary surfactant.^5, 6, 7^ Studies also reported that AT-II cells are potential cancer stem cells that lead to the development of non-small cell lung cancer (NSCLC),^8, 9^ suggesting potential relation between SLC34A2 and cancer. Increasing but conflicting studies indicate that SLC34A2 has a critical role in lung cancer: Kiyamova *et al.*^10^ and Kopantzev *et al.*^11^ reported decreased expression of SLC34A2 in NSCLC comparing with corresponding normal tissue. Wang *et al.*^12^ further demonstrated the suppressive effects of SLC34A2 on tumorigenesis and progression, which may be associated with the downregulation of related protein in PI3K/Akt and Ras/Raf/MEK signal pathway. More recently, miR-410 is reported to positively contribute to the tumorigenesis and development of NSCLC by downregulating *SLC34A2*.^13^ However, Hong *et al.*^14^ showed downregulating SCL34A2 successfully suppressed lung cancer growth and decreased cancer cell proliferation and angiogenesis, and facilitated apoptosis. Another study reported that SLC34A2 regulated Bmi1 to promote tumorigenic and self-renewal potential of CD166(+) lung cancer stem cell-like cells through Wnt/*β-catenin* pathway.^15^ Unlike the situation in lung cancer, it is widely accepted that SLC34A2 is upregulated in ovarian cancer, especially in well-differentiated tumors.^10, 16, 17^ Furthermore, enhanced SLC34A2 expression was reported to be correlated with chemo-response and survival of breast cancer patients, and downregulation of SLC34A2 could sensitize breast cancer stem cells to doxorubicin.^18^ Meanwhile, SLC34A2 is downregulated in renal cell carcinoma because of promoter methylation.^19^ Thus, SLC34A2 is dysregulated and acts as either an oncogene or tumor-suppressor gene in different cancers. Up to date, however, the expression dynamics of SLC34A2 in BC and its clinicopathologic/prognostic significance have not been elucidated. Therefore, it is of great significance to explore the role of SLC34A2 in BC.

In this study, we aimed to identify the expression status of SLC34A2 in BC cell lines and tissues. We next proceeded to investigate the SLC34A2 expression pattern in two different cohorts of BC patients who underwent radical cystectomy. Furthermore, the function of SLC34A2 to the pathogenesis and progression of BC were explored. We report for the first time that SLC34A2, a target of miR-214, is obviously upregulated in BC, and serves as an independent prognostic indicator for BC patients. Moreover, SLC34A2 promotes BC cell proliferation and tumor growth both *in vitro* and *in vivo*, which is achieved via upregulating expression level and transcriptional activity of c-Myc.

## Results

### Elevated expression of SLC34A2 in BC

**Figure 1:**
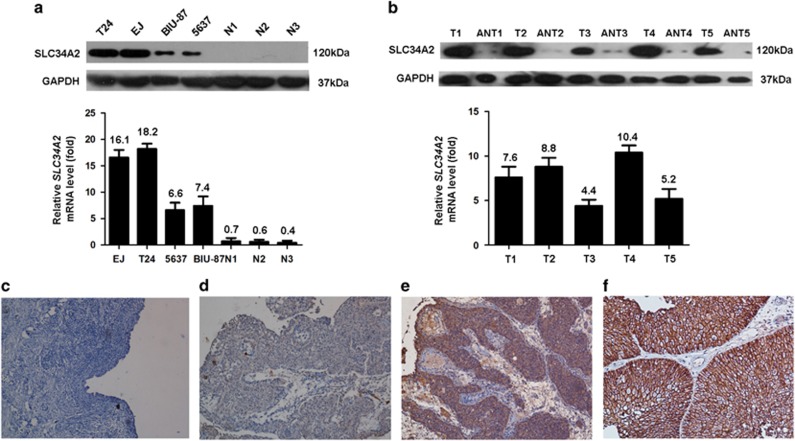
Western blotting, quantitative RT-PCR and IHC assay of the expression of SLC34A2 in BC cell lines and tissues. (**a**and**b**) Western blotting (upper panel) and quantitative RT-PCR (lower panel) assay of SLC34A2 expression in (**a**) four BC cell lines and three normal bladder tissues (N), and in (**b**) five pairs of matched BC (T) and adjacent non-neoplastic bladder tissue (ANT). The average expression level of *SLC34A2* in N or ANT was used as a loading control in quantitative RT-PCR assay. (**c-f**) Representative image of SLC34A2 IHC staining in BC tissues: negative staining in normal bladder tissue (**c**), and weak (**d**), moderate (**e**) and strong (**f**) staining in BC tissues, respectively. Images are presented at × 200 magnification

To investigate the expression status of SLC34A2 in BC, western blotting and quantitative RT-PCR analyses were performed in four BC cell lines (EJ, T24, 5637 and BIU-87), three normal bladder tissues and five fresh BC tissues paired with their adjacent non-neoplastic bladder tissues (ANTs). As shown in Figure 1a, SLC34A2 were significantly upregulated in all four BC cell lines compared with normal bladder tissues both on protein and mRNA levels. Similarly, SLC34A2 were considerably higher in BC tissue specimens when compared with their paired ANTs (Figure 1b). We then conducted immunohistochemical (IHC) assays in two independent cohorts of BC tissue slices to determine the protein level of SLC34A2. Representative staining of SLC34A2 in BC tissue is shown in Figures 1c and f. The positive staining is observed in the membrane and cytoplasm of BC cells. Analogously, IHC assays revealed a similar expression pattern of SLC34A2 in BC and the corresponding ANTs, where SLC34A2 was overexpressed in BC tissue.

### Selection of cutoff score for high expression of SLC34A2 in BC

**Figure 2:**
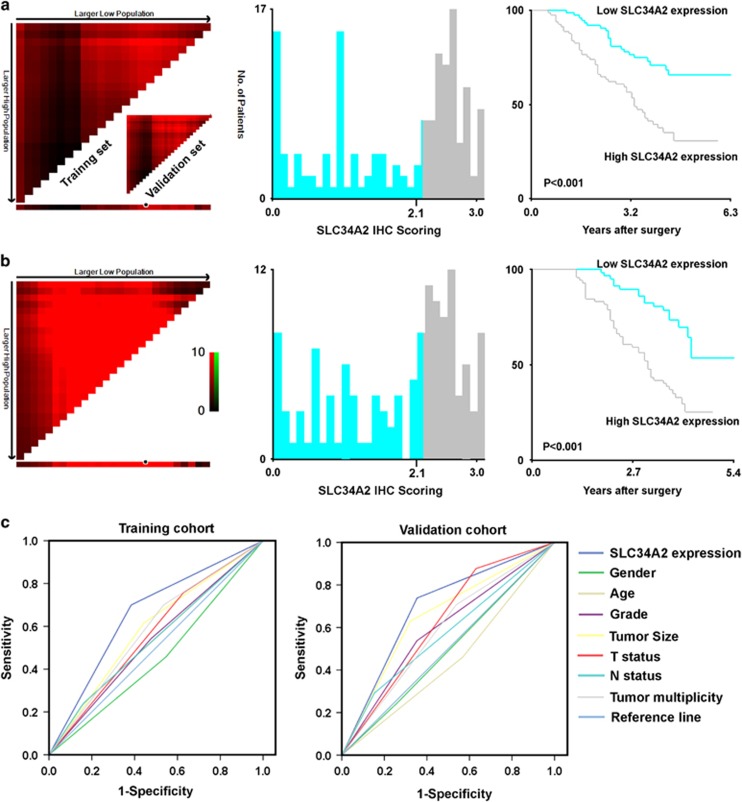
The selection of optimal cutoff value for SLC34A2 IHC score in two different cohorts of BC samples. (**a**) X-tile analysis was conducted on patient data from the training cohort, equally subdivided into training and validation subsets. X-tile plots of training sets are displayed in the left panels, with matched validation sets in the smaller inset. The plot showed the *χ*^2^ log-rank values created when the cohort was divided into two populations. The cut point was demonstrated on a histogram of the entire cohort (middle panels) and a Kaplan–Meier plot (right panels). *P-*values were defined by using the cut point derived from a training subset to parse a separate validation subset. SLC34A2 expression was divided at the optimal cut point (for high expression, *n*=82; for low expression, *n*=74), where the plot achieves highest significance (with positive staining of SLC34A2; *P*<0.001). (**b**) The optimal cut point for SLC34A2 expression determined by X-tile plot of the testing cohort was applied to the validation cohort (for high expression, *n*=71; for low expression, *n*=59) and reached high statistical significance (*P*<0.001). (**c**) ROC curve analysis for different clinicopathological features and SLC34A2 expression was performed to evaluate the survival status. Left panel: age (AUC=0.455; *P*=0.338), gender (AUC=0.539; *P*=0.406), grade (AUC=0.533; *P*=0.478), tumor size (AUC=0.586; *P*=0.064), T statue (AUC=0.565; *P*=0.166), N statue (AUC=0.540; *P*=0.390), tumor multiplicity (AUC=0.583; *P*=0.046) and SLC34A2 expression (AUC=0.658; *P*=0.001) implied statistical associations with survival in the training cohort; Right panel: age (AUC=0.446; *P*=0.289), gender (AUC=0.492; *P*=0.880), grade (AUC=0.592; *P*=0.069), tumor size (AUC=0.654; *P*=0.002), T statue (AUC=0.623; *P*=0.015), N statue (AUC=0.569; *P*=0.173), tumor multiplicity (AUC=0.585; *P*=0.050) and SLC34A2 expression (AUC=0.692; *P*<0.001) were used to test the survival status in validation cohort

To assess the statistical significance and avoid arbitrary cut point selection, we applied the X-tile program to obtain cutoff scores for SLC34A2 expression. Using the X-tile plots for the training cohort, we divided this cohort into low and high populations based on a cutoff score of ‘2.1' for SLC34A2 IHC staining (Figure 2a). This optimal cut point determined by the training cohort was then applied to the validation cohort, which identified the cutoff score to have high statistical significance again (*P*<0.001; Figure 2b). According to this cut point, high SLC34A2 expression was observed in 82 of 156 (52.6%) BCs and 8 of 50 (16%) adjacent non-neoplastic bladder tissues (ANTs) in the training cohort (*P*<0.001). In the validation cohort, high SLC34A2 expression was observed in 71 of 130 (54.6%) BC cases.

The rates of high expression of SLC34A2 in BC with respect to clinicopathologic features were detailed in Table 1. We observed that high expression of SLC34A2 was positively associated with larger tumor size (*P*=0.017; *P*=0.012, respectively) and advanced T status (*P*<0.001; *P*=0.008, respectively) both in training and validation cohorts of BC cases (Table 1). These data suggested that the expression of SLC34A2 increases with BC progression.

### Elevated SLC34A2 expression predicted poor prognosis

To further confirm the prognostic value of SLC34A2 expression and clinicopathological features in BC, we applied receiver operating characteristic (ROC) curves to test patient survival status in our study. According to the ROC curve analysis, SLC34A2 was found to be a promising predictor for survival status both in training (area under the curve (AUC)=0.658; *P*=0.001) and validation cohort (AUC=0.692; *P*<0.001; Figure 2c). Furthermore, our univariate and multivariate analysis showed that high expression of SLC34A2 was an independent risk factor for adverse overall survival (OS) in both training (hazard ratio (HR): 3.006; 95% confidence interval (CI): 1.746–5.172, *P*<0.001; Table 2) and validation cohorts (HR: 2.364, 95% CI: 1.333–4.1934, *P*=0.003; Table 2). In addition, survival analysis illustrated that SLC34A2 expression could significantly stratify OS in a subset of BC patients with different age, gender, T status, N status, overall clinical stage, tumor grade and tumor size (Supplementary Figure 1).

### Effects of SLC34A2 on cell proliferation *in vitro*

**Figure 3:**
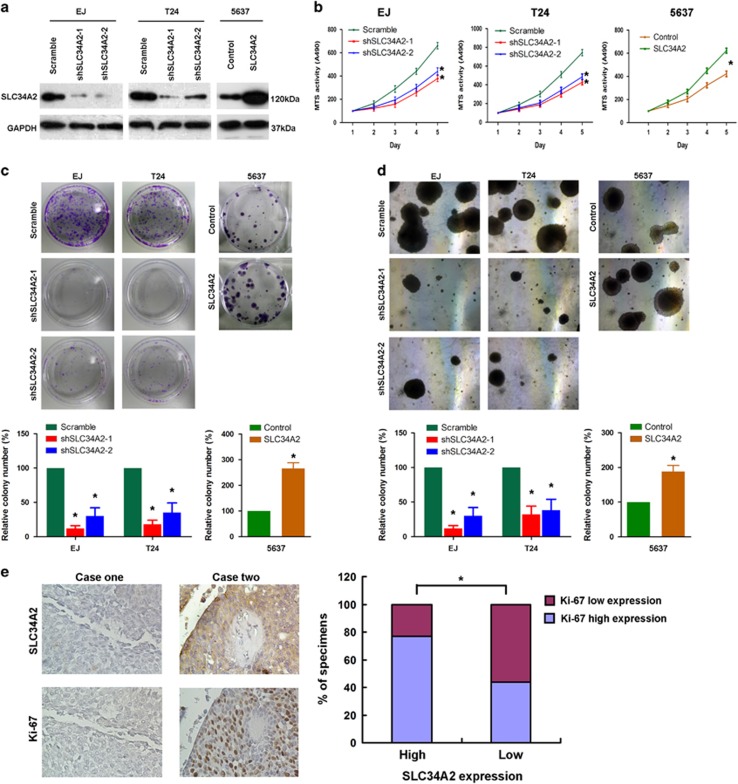
Effects of SLC34A2 on cell proliferation *in vitro*. (**a**) The protein expression of SLC34A2 analyzed by western blotting in shSLC34A2 transduced EJ and T24 cells compared with scramble controls, and SLC34A2 stably overexpressed 5637 cells compared with negative control cells. (**b** and **c**) EJ and T24 cells with reduced SLC34A2 showed significant growth inhibition compared with scramble controls, and 5637 cells with upregulated SLC34A2 proved growth promotion compared with controls, as determined by (**b**) MTT assays and (**c**) colony formation assays. (**d**) Downregulation of SLC34A2 greatly suppressed the anchorage-independent growth of both EJ and T24 cells, but upregulation of SLC34A2 significantly accelerated the malignancy transforming growth of 5637 cells. Colonies in (**c**) and (**d**) larger than 0.1mm diameter were quantified after 14 days of culture. (**e**) Left panel: expression of SLC34A2 is positively associated with Ki-67 in clinical BC specimens. Two representative cases are shown. Right panel: percentage of specimens showing low or high SLC34A2 expression in relation to the expression levels of Ki-67. *P<0.05

Two short hairpin RNAs (shRNAs) specifically directed against *SLC34A2* (shSLC34A2) were induced into EJ and T24 cell lines, which exhibit high SLC34A2 expression, whereas exogenous SLC34A2 was stably introduced into 5637 cell, which shows relative low SLC34A2 expression (Figure 3a). Next, MTT assays and colony formation assays were used to assess the effects of SLC34A2 on cell viability and proliferation ability. EJ and T24 cells transfected with shSLC34A2 showed significant growth inhibition compared with scramble controls (*P*<0.001), and overexpression of SLC34A2 promoted the growth of 5637 cells compared with negative control cells (*P*<0.001; Figure 3b). Fewer and smaller colonies were observed in shSLC34A2 treated cells when compared with scramble controls (*P*<0.001); whereas overexpression of SLC34A2 resulted in more and larger colonies compared with control cells (*P*<0.001; Figure 3c). Furthermore, we use anchorage-independent growth ability assay of soft agar to determine the ability of SLC34A2 in malignancy transforming. Results showed that downregulation of SLC34A2 greatly suppressed the anchorage-independent growth of both EJ and T24 cells (*P*<0.001), but upregulation of SLC34A2 significantly accelerated the malignancy transforming growth of 5637 cells (*P*<0.001; Figure 3d). Taken together, these results suggested the pro-proliferative role of SLC34A2 in human BC cells *in vitro.* We further examined the relationship between SLC34A2 and Ki-67 expression in BC tissues, as the latter is a marker for cellular proliferation. We observed that BC cases with high SLC34A2 expression also exhibited strong Ki-67 staining signals, whereas those with low levels of SLC34A2 displayed weak Ki-67 staining (Figure 3e). The chi-square testing showed a significant correlation between SLC34A2 expression and the Ki-67 labeling index in BC (*P*<0.001; Figure 3e).

### Effects of SLC34A2 on tumor growth *in vivo*

**Figure 4:**
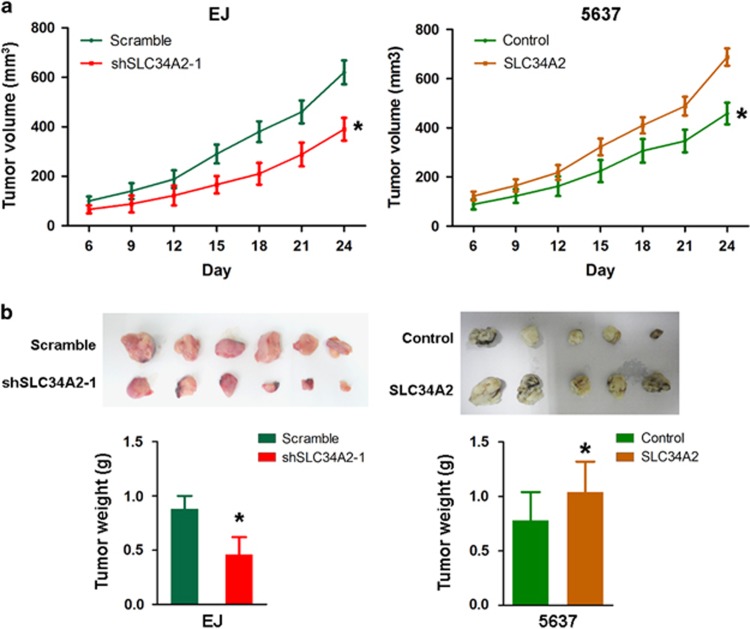
Effects of SLC34A2 on tumor growth *in vivo*. (**a**) The xenograft tumor volumes of nude mice injected with EJ/scramble cells or EJ/shSLC34A2 cells (*n*=6), and that of nude mice injected with 5637/control cells or 5637/SLC34A2 cells (*n*=5). (**b**) The average tumor weight in nude mice injected with EJ/shSLC34A2 cells were significantly decreased compared with the EJ/scramble group (*n*=6), whereas 5637 cells with overexpressed SLC34A2 largely increased the tumor burden (*n*=5). *P<0.05

To further confirm the effect of SLC34A2 required for BC tumor growth *in vivo*, xenograft tumor model assays were conducted by injecting EJ-shSLC34A2/scramble cells and 5637-SLC34A2/control cells into the dorsal flank of nude mice subcutaneously. The EJ/shSLC34A2 cells grew at a much slower rate than EJ/scramble cells, whereas SLC34A2 overexpression accelerated the xenograft tumor growth (*P*<0.001; Figure 4a). Furthermore, the average weight of tumor was significantly lower in the SLC34A2 depletion group compared with the EJ/scramble group (*P*<0.001), whereas overexpression of SLC34A2 in 5637 cells largely increased the tumor burden (*P*<0.001; Figure 4b). Collectively, this gave direct evidence of SLC34A2's role in promoting BC carcinogenesis *in vivo*.

### Depletion of SLC34A2 suppressed the expression and transcription capacity of c-Myc

**Figure 5:**
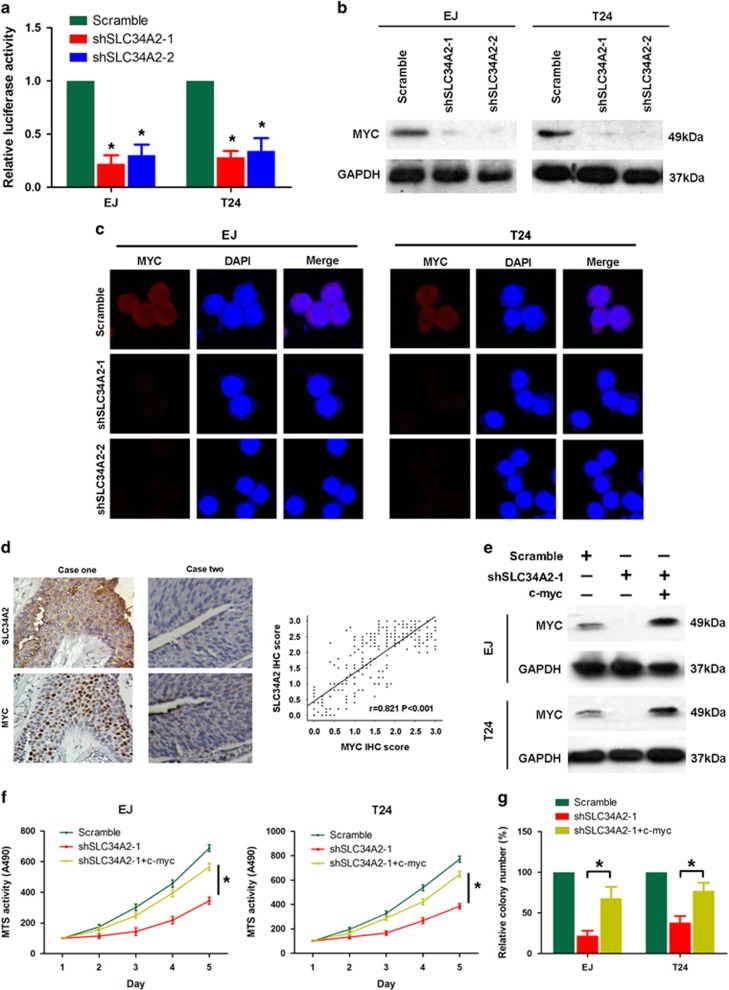
Depletion of SLC34A2 suppressed the expression and transcription capacity of c-Myc. (**a**) The luciferase reporter activities of c-Myc were remarkably decreased in SLC34A2-silenced EJ and T24 cells. (**b** and **c**) In SLC34A2 stably reduced EJ and T24 cells, the protein expression of c-Myc was decreased as determined by (**b**) western blotting analysis and (**c**) immunofluorescence staining. (**d**) Left panel: expression of SLC34A2 is positively associated with c-Myc in clinical BC specimens. Two representative cases are shown. Right panel: Pearson's correlation coefficients analysis showed that SLC34A2 positively correlated with c-Myc expression in BC samples. (**e**) In both EJ and T24 cells, endogenous c-Myc were greatly downregulated in the presence of shSLC34A2–1, the suppression was reversed by inducing exogenous c-Myc. (**f-g**) The inhibition of cell viability and proliferation ability by shSLC34A2 was significantly compromised by treatment with exogenous c-Myc, as determined by (**f**) MTT assays and (**g**) colony formation assays. *P<0.05

As c-Myc is a critical transcription factor, which binds target DNA sequences to regulate transcription of genes involved in cell growth and proliferation, we further explored the transcription capacity of c-Myc using dual-luciferase reporter assay in the presence of shSLC34A2. As shown in Figure 5a, the relative luciferase reporter activity was remarkably decreased in SLC34A2-silenced cells. Interestingly, we also noticed the depletion of SLC34A2 by shRNAs suppressed the expression of c-Myc simultaneously in both EJ and T24 cells by the western blotting (Figure 5b) and immunofluorescence analysis (Figure 5c). Furthermore, our IHC analysis illustrated that expression levels of SLC34A2 and c-Myc proteins were positively correlated in the total BC cases enrolled (*r*=0.821, *P*<0.001; Figure 5d). These data suggest that c-Myc is closely linked with SLC34A2, it may involve in SLC34A2-related cell growth and proliferation.

To confirm these results, exogenous c-Myc was induced into EJ/T24 shSLC34A2–1 cells, in which endogenous c-Myc were greatly downregulated in the presence of shSLC34A2–1 (Figure 5e). As shown in Figures 5f and g, the inhibition of cell viability and proliferation ability by shSLC34A2 was significantly compromised by treatment with exogenous c-Myc, as determined by MTT assays and colony formation assays. Taken together, these data indicate that SLC34A2 enhances tumor cell growth and proliferation through upregulation of c-Myc, which is a well-known oncogenic factor.

### MiR-214 is a negative regulator of SLC34A2

**Figure 6:**
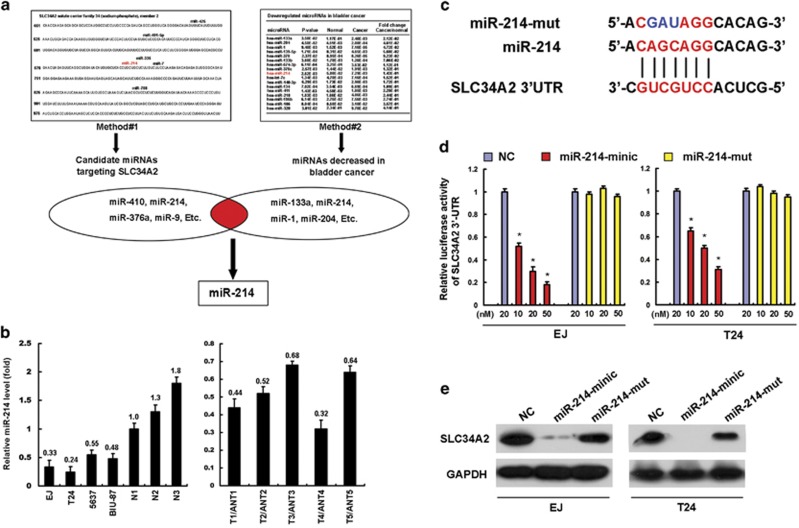
MiR-214 is a negative regulator of SLC34A2. (**a**) Illustration of screening miRNAs regulating *SLC34A2*: Method #1 used the prediction algorithm of *MICRORNA.ORG* identified 23 miRNAs, which hold potential as SLC34A2 suppressors. Method #2 used miRNA profiling and identified 17 miRNAs, which were downregulated significantly in BC. Then, we overlapped the results of two methods and singled out miR-214 as a potential regulator. (**b**) Quantitative RT-PCR assay of miR-214 expression in four BC cell lines and three normal bladder tissues (N) (left panel), and in five pairs of matched BC (T) and adjacent non-neoplastic bladder tissue (ANT) (right panel). The average expression level of miR-214 in N or ANT was used as a loading control in quantitative RT-PCR assay. (**c**) Predicted miR-214 target sequence in 3′-UTR of *SLC34A2* (SLC34A2 3′-UTR) and mutant miRNA containing three altered nucleotides in the seed sequence of miR-214 (miR-214-mut). (**d**) Luciferase assay of pGL3-SLC34A2 3′-UTR reporters co-transfected with increasing amounts (10, 20 and 50 nM) of miR-214 mimic or miR-214-mut in EJ and T24 cell lines. (**e**) Western blotting analysis demonstrates that miR-214 transfection markedly decreased SLC34A2 protein levels of EJ and T24 cells, whereas miR-214-mut exerted no inhibition effect. *P<0.05

As post-translational regulation such as microRNA (miRNA) had critical role in protein regulation, we sought to explore whether the dysregulation of miRNAs was responsible for the upregulation of SLC34A2 in BC. We first conducted bioinformatics analyses and overlapped the predicted miRNA regulators with downregulated miRNAs from miRNA expression profiles of BC.^20^ The result showed that miR-214 was singled out as a potential regulator of SLC34A2 (Figure 6a).

Next, our quantitative RT-PCR analyses showed that miR-214 was indeed downregulated in all four BC cell lines and five fresh BC tissues examined (Figure 6b). To verify the hypothesis that downregulation of miR-214 was responsible for the upregulation of SLC34A2 in BC, we constructed miR-214 mutant(miR-214-mut), which mismatched the 3'-UTR of *SLC34A2* (Figure 6c). Results of the luciferase reporter assay showed that miR-214 overexpression decreased the luciferase activity of the SLC34A2 3'-UTR, whereas the miR-214-mut failed to show an inhibitory effect on the luciferase expression (Figure 6d). Furthermore, western blotting assays proved that miR-214 greatly downregulated the protein level of SLC34A2, whereas miR-214-mut exerted none of the above effects (Figure 6e). These data collectively provided evidence that miR-214 directly suppresses *SLC34A2* expression and decreased miR-214 contributes to SLC34A2 overexpression in BC.

## Discussion

According to National Central Cancer Registry of China 2015 annual report, the incidence of BC in male cancers was 7.68/10^5^ in 2011, and the mortality rate was 3.03/10^5^, which ranked sixth and tenth amount all cancers, respectively.^21^ Chinese Bladder Cancer Consortium reported that surgical therapies especially transurethral resection and radical cystectomy are generally applied for non-muscle invasive BC (NMIBC) and muscle invasive BC (MIBC). About 70% of the NMIBC patients accepted chemotherapy instillation, and 20.3% MIBC patients accepted neo-adjuvant or adjuvant chemotherapy. However, the long-term prognosis of BC patients remains unsatisfactory. Five-year cumulative intravesicle recurrence is about 35% in NMIBC, whereas MIBC patients have a 5-year overall survival of about 60%.^22^ Thus, there is an urgent need to identify biomarkers, which can be used to define the malignancy potential of BC or as potential therapeutic targets.

In this study, we sought to explore the role of SLC34A2 in human BC. We report, for the first time, that the expression of SLC34A2 is pervasively upregulated in BC cell lines and tissues. Furthermore, high expression of SLC34A2 in BC is a strong marker for poor prognosis. These results indicate that SLC34A2 has a critical role in BC carcinogenesis and may facilitate highly malignancy transforming. As a widely accepted fact, advanced stage of tumor and recurrence are major causes of cancer-related death. Notably, our analyses demonstrated that high level of SLC34A2 was significantly correlated with advanced T stage and larger tumor size. In consistent, our *in vitro* and *in vivo* studies showed that overexpression of SLC34A2 increased cellular viability and proliferation ability, but the deletion of SLC34A2 repressed these abilities. We take this promotion of tumor growth as a potential underlying mechanism in SLC34A2-mediated BC carcinogenesis. Thus, SLC34A2 expression may be a potential marker for malignancy of BC and an intervention target for treatment, that is, it implies that patients with high expression of SLC34A2 may need chemoradiation or molecular target therapy for tumor control after radical cystectomy, whereas those with low expression of SLC34A2 can avoid unnecessary treatments except for surgery. A mouse monoclonal antibody MX35 targeting SLC34A2 protein developed by Kiyamova *et al.*^23, 24^ have provided data on the pattern of SLC34A2 expression and cellular localization in human breast, lung and ovarian cancers.^10, 17^ However, the origin of murine limited the antibody's full therapeutic potential. To overcome this impediment, a humanized antibody version named Rebmab200 was then developed. Analyses of Rebmab200 antibody demonstrated that it had strong reactivity with the tested tumor types but little or no reactivity with the tested normal tissues. A translational phase I clinical trials of Rebmab200 are now in progress.^25^ Thus, targeted therapy with SLC34A2 antibody in BC may be a viable option and deserve further testing in the near future.

c-Myc, one of the most studied oncogenes, is involved in several malignant cellular processes, such as cell growth and proliferation.^26, 27^ Analyses showed that in silenced SLC34A2 cells, the expression and transcription capacity of c-Myc were remarkably decreased. When exogenous c-Myc was induced into EJ/T24 shSLC34A2–1 cells, the inhibition of cell viability and proliferation ability exerted by decreased SLC34A2 was significantly compromised. Thus, we conclude that SLC34A2 enhances tumor cell growth and proliferation through upregulation of c-Myc, which is a well-known oncogenic factor. It expands another alternative strategy to inhibit cancer progression initiated by SLC34A2 in BC, that is, to target the signaling mechanisms, by the use of c-Myc inhibitors.

As the expression of SLC34A2 is pervasively upregulated in BC cell lines and tissues, one critical question was then raised: what is the mechanism by which SLC34A2 is upregulated? A class of miRNAs has been proved as important regulators of gene expression,^28, 29^ in this study with the help of bioinformatics analyses and miRNA expression profiles of BC cells,^20^ we revealed that miR-214 directly suppresses SLC34A2 expression and decreased miR-214 contributes to SLC34A2 overexpression in BC. This finding is based on evidence as below: first, miR-214 has a conserved binding site in the 3'-UTR of *SLC34A2*; second, the luciferase activity of *SLC34A2* 3'-UTR reporter is specifically responsive to increased miR-214 but non-reactive to miR-214-mut; third, the overexpression of miR-214 reduced the expression of SLC34A2; and fourth, miR-214 is downregulated in BC cells. Therefore, upregulation of miR-214, such as using miR-214 mimics, is an optional strategy to suppress BC, which has high level of *SLC34A2*.

In conclusion, we report for the first time that SLC34A2, target of miR-218, is overexpressed pervasively in BC, and it is correlated with poor prognosis, advanced T stage and larger tumor size. Furthermore, the deletion of SLC34A2 repressed carcinogenesis of BC cells both *in vitro* and *in vivo* through inhibiting the expression and transcription capacity of c-Myc. These findings suggest that SLC34A2 has role in the development and progression of human BC, which render SLC34A2 a potential prognostic marker and may serve as a novel therapeutic target in BC patients.

## Materials and Methods

### Cell lines and stable cell line construction

The human BC cell lines EJ, T24, 5637 and BIU-87 were grown in RPMI-1640 medium according to American Type Culture Collection (ATCC, Manassas, VA, USA) instructions with 10% fetal bovine serum (Invitrogen, Carlsbad, CA, USA). All cell lines were cultured in a humidified chamber with 5% CO_2_ at 37 °C. All experiments were performed in cultures that were 70–80% confluent, the cells in log-phase growth.

The two shRNA used to repress *SLC34A2* expression and the coding sequences of SLC34A2 expression vector are purchased from GeneCopoeia (Guangzhou, China). Vector construction, lentivirus production and infection were conducted as previously described.^30^

### BC sample selection

Tumor tissues collected from 156 BC patients were formalin-fixed and paraffin-embedded (FFPE). These patients were enrolled from the Department of Urology at The First Affiliated Hospital of Sun Yat-Sen University and The Hospital of Guangdong Province where they underwent radical cystectomy between January 2003 and December 2008. Fifty paraffin-embedded specimens of ANT from the same BC patients were used as controls. In addition, five fresh pairs of BC tissues and matched ANTs, as well as three specimens of normal bladder tissues from patients without BC-related disease were frozen and stored in liquid nitrogen until further use. In parallel, we obtained another independent validation cohort of FFPE samples from 130 BC patients who underwent radical cystectomy from the Department of Urology of Sun Yat-Sen University Cancer Center (Guangzhou, China) between March 2003 and February 2008. All the BC patients enrolled had no radio-/chemical therapy histories before surgery. Clinical data were collected from raw case reports and staged according to the World Health Organization and the sixth edition of pTNM classification of the Union for International Cancer Control (UICC, 2002). All the patients were followed up on regular basis and the OS was defined as the time from surgery to the date of the death date or when censured at the latest date if patients were still alive. The clinicopathologic characteristics of the patients in each cohort are summarized in Table 1. Samples were obtained after given informed consent in accordance with the approval by institutional ethical review board.

### IHC staining assays and selecting the optimal cutoff value

IHC staining assays were performed on 4-*μ*m-thick FFPE sections. Briefly, sections were de-waxed, boiled in retrieval buffer solution for antigen recovery and then incubated with primary antibody overnight at 4 °C, followed by the Dako Real Envision Kit (Dako, Glostrup, Denmark), which was used to visualize protein expression. The intensity of staining in tumor cells was scored by two pathologists independently. First, tumor cells in five fields were randomly selected and scored based on the percentage of positively stained cells (0–100%). Then, the positive staining cells of different intensities were assessed: 0, no staining; 1, weak (light yellow); 2, moderate (yellow brown); 3, strong (brown). Finally, a semiquantitative IHC score ranging from 0 to 3 was calculated by multiplying the percentage of positively stained cells with each category of staining intensity.

The optimal cutoff IHC score of SLC34A2 expression was selected using X-tile plots (Yale University School of Medicine, New Haven, CT, USA).^31^ At first, we applied the X-tile program software to generate the optimal SLC34A2 IHC cutoff score to accurately classify patients according to clinical outcome in the training cohort. In the validation cohort, the cutoff score derived from X-tile analysis was investigated to exam the association of SLC34A2 expression with patients overall survival. X-title data were presented in a right triangular grid where each point represents a different cut point. The intensity of the color of each cutoff point represents the strength of the association. The X-tile program can automatically select the optimal data cut point according to the highest chi-square value (minimum *P*-value) defined by Kaplan–Meier survival analysis and log-rank test.^32^ X-tile plots were performed with X-tile software version 3.6.1 (Yale University School of Medicine, New Haven, CT, USA).

### Statistical analysis

All *in vitro* experiments were repeated at least three times. Statistical analyses were performed using the SPSS Standard version 16.0 software package (SPSS Inc., Chicago, IL, USA). ROC curve analysis was conducted to evaluate the predictive value of the parameters. Kaplan–Meier and log-rank tests were used to analyze patient survival, and *χ*^2^ tests were used to analyze the associations between SLC34A2 expression and clinical–pathological parameters. Comparisons between groups for statistical significance were performed with a two-tailed paired Student's *t*-test. Bivariate correlations between study variables were calculated by Pearson's correlation coefficients. Data are presented as mean±S.D. *P*-values <0.05 were considered statistically significant.

Other Materials and methods are available in the Supplementary Materials and Methods.

## Figures and Tables

**Table 1:** Association of SLC34A2 expression with patient's clinicopathological features in BC

	**SLC34A2 expression**							
		**Training cohort**				**Validation cohort**		
**Variables**	**Case**	**Low expression**	**High expression**	***P*****-value**	**Case**	**Low expression**	**High expression**	***P*****-value**
*Age*		74	82			59	71.000	
⩽57^a^	77	34 (44.2%)	43 (55.8%)		63	26 (41.3%)	37 (58.7%)	
>57	79	40 (50.6%)	39 (49.4%)	0.418	67	33 (49.3%)	34 (50.7%)	0.361
								
*Gender*								
Male	127	61 (48.0%)	66 (52.0%)		97	42 (43.3%)	55 (56.7%)	
Female	29	13 (44.8%)	16 (55.2%)	0.755	33	17 (51.5%)	16 (48.5%)	0.413
								
*WHO grade*								
G1/2	77	32 (41.6%)	45 (58.4%)		72	35 (48.6%)	37 (51.4%)	
G3	79	42 (53.2%)	37 (46.8%)	0.147	58	24 (41.4%)	34 (58.6%)	0.410
								
*Tumor size (cm)*								
⩽5^b^	75	43 (57.3%)	32 (42.7%)		68	38 (55.9%)	30 (44.1%)	
>5	81	31 (38.3%)	50 (61.7%)	0.017^c^	62	21 (33.9%)	41 (66.1%)	0.012^c^
								
*T status*								
T1/2	49	35 (71.4%)	14 (28.6%)		32	21 (65.6%)	11 (34.3%)	
T3/4	107	39 (36.4%)	68 (63.6%)	<0.001^c^	98	38 (38.8%)	60 (61.2%)	0.008^c^
								
*N status*								
N0	125	62 (49.6%)	63 (50.4%)		101	48 (47.5%)	53 (52.5%)	
N1/2	31	12 (38.7%)	19 (61.3%)	0.277	29	11 (37.9%)	18 (62.1%)	0.360
								
*Tumor multiplicity*								
Unifocal	61	24 (39.3%)	37 (60.7%)		49	27 (55.1%)	22 (44.9%)	
Multifocal	95	50 (52.6%)	45 (47.4%)	0.105	81	32 (39.5%)	49 (60.5%)	0.083

a Mean age

b Mean tumor size

c Statistically significant difference

**Table 2:** Univariate and multivariate analysis of SLC34A2 expression and various clinicopathological parameters in training and validation cohort patients with BC

	**Training cohort**				**Validation cohort**			
**Variables**	**Case**	**HR**	**95% CI**	***P*****-value**	**Case**	**HR**	**95% CI**	***P*****-value**
*Univariate analysis*								
Age								
⩽60^a^	77	1.0			63	1.0		
>60	79	0.866	0.541–1.386	0.549	67	0.745	0.457–1.214	0.238
Gender								
Female	127	1.0			97	1.0		
Male	29	1.366	0.782–2.387	0.273	33	0.99	0.563–1.742	0.973
WHO grade								
G1/2	77	1.0			72	1.0		
G3	79	1.562	0.915–2.665	0.102	58	1.678	1.029–2.737	0.038^b^
Tumor size (cm)								
⩽5^c^	75	1.0			68	1.0		
>5	81	1.838	1.133–2.981	0.014^b^	62	2.487	1.497–4.129	<0.001^b^
T status								
T1/2	49	1.0			32	1.0		
T3/4	107	1.986	1.148–3.434	0.014^b^	98	2.929	1.395–6.147	0.005^b^
N status								
N0	125	1.0			101	1.0		
N1	31	1.639	0.948–2.833	0.077	29	1.764	1.032–3.016	0.038^b^
Tumor multiplicity								
Unifocal	61	1.0			49	1.0		
Multifocal	95	1.717	1.028–2.866	0.039^b^	81	1.721	1.007–2.941	0.047^b^
SLC34A2 expression								
Low expression	74	1.0			59	1.0		
High expression	82	2.902	1.737–4.850	<0.001^b^	71	3.34	1.915–5.828	<0.001^b^
								
*Multivariate analysis*								
Tumor size (cm)								
⩽5^c^	75	1.0			68	1.0		
>5	81	1.642	0.997–2.702	0.051	62	2.269	1.313–3.920	0.003^b^
T status								
T1/2	49	1.0			32	1.0		
T3/4	107	1.849	1.014–3.374	0.045^b^	98	2.245	1.057–4.768	0.035^b^
N status								
N0	125	1.0			102	1.0		
N1	31	1.542	0.870–2.734	0.138	28	1.978	1.125–3.477	0.018^b^
Tumor multiplicity								
Unifocal	61	1.0			49	1.0		
Multifocal	95	2.029	1.206–3.415	0.008^b^	81	1.382	0.799–2.390	0.247
WHO grade								
G1/2	77	1.0			72	1.0		
G3	79	1.809	1.092–2.997	0.021^b^	58	1.261	0.756–2.103	0.375
SLC34A2 expression								
Low expression	74	1.0			59	1.0		
High expression	82	3.006	1.746–5.172	<0.001^b^	71	2.364	1.333–4.1934	0.003^b^

Abbreviations: CI, confidence interval; HR, hazard ratio

Cox proportional hazard regression model, enter

a Mean age

b Statistically significant difference

c Mean tumor size.
