# Supplementary material for: Overexpression of SLC34A2 is an independent prognostic indicator in bladder cancer and its depletion suppresses tumor growth via decreasing c-Myc expression and transcriptional activity
Source: Cell Death Dis. 2017 Feb 2;8(2):e2581–. doi: 10.1038/cddis.2017.13 (PMC5386463; doi:10.1038/cddis.2017.13)
Supplement: Supplementary Information [file cddis201713x1.doc]

**Supplementary Material and Methods**

**RNA extraction, reverse transcription, and quantitative RT-PCR**

Total RNA from cultured cells and fresh tissues was isolated using TRIzol reagent (Invitrogen, Carlsbad, CA). Complementary DNA was reverse-transcribed from 2 μg of total RNA using an oligo-dT primer after RNase-free DNase I treatment (Promega). We applied an ABI-7900HT fast real-time system (Applied Biosystems, Foster City, CA) to determine *SLC34A2* mRNA levels in each sample with SsoFast EvaGreen Supermix (Bio-Rad Laboratories). Gene expression was normalized to the housekeeping gene glyceraldehyde 3-phosphate dehydrogenase(GAPDH) and compared with the normal controls. The primers used for *SLC34A2*and *GAPDH* were purchased from GeneCopoeia (Guangzhou, China).

**MTT assay**

To detect relative cell proliferation abilities, 20 µL of 3-(4,5-dimethylthiazole-2-yl)-2,5-biphenyl tetrazolium bromide (MTT, 5 mg/mL) was added per day to 96-well plates till the cells were seeded for five days. The plates were then incubated at 37°C for 4 hours, followed by solubilized of the product in 100 µL of DMSO (Sigma Aldrich). Absorbance was measured at 490 nm using an ELISA reader. The proliferative abilities were expressed as the relative percentage of cell numbers on the 1^st^ day.

**Western blotting analyses**

Equal amount of denatured cell protein was separated by SDS-PAGE gels and transferred to a polyvinylidene fluoride membrane (Roche, Swiss). Membranes were incubated with primary antibodies against: SLC34A2 (Cell Signaling Technology, USA), c-Myc, and GAPDH (Abcam, Cambridge, UK), followed by proper secondary antibodies linked with HRP. GAPDH served as loading controls. An ECL Western blot detection kit (ThermoFisher Scientific) was used for detection.

**Colony formation assay and anchorage-independent growth ability assay**

For the colony formation assay, 500 cells of each cell group were plated into 6-well plates with complete medium. After 14 days, visible colonies were fixed and stained with 0.1% crystal violet. Colony number was then calculated.

In the anchorage-independent growth ability assay, 500 cells of each group were tripsinized and resuspended with 2 mL complete medium plus 0.3% agar (Sigma, Saint Louis, MI). Then, the cell-agar mixture was placed on top of a bottom layer made of complete medium and 1% agar. After 14 days, colonies larger than 0.1mm were counted.

**Xenograft tumor model assay**

Six weeks old, male severe combine immunodeficient (SCID-Biege) mice were randomized into four groups. To investigate the impact of SLC34A2 on proliferation *in vivo*, EJ shSLC34A2-1/scramble cells or 5637 SLC34A2/control cells were injected subcutaneously into the dorsal flank of each nude mice, respectively (3×10^6^ cells) on day 1 morning. Mice were followed-up every three days to measure tumor sizes with calipers. Tumor volume was calculated as: Volume=largest diameter × (smallest diameter)^2^ /2. 24 days after injection, mice were killed and tumors were weighed and photographed. All animal studies were conducted with the approval of the Sun Yat-sen University Institutional Animal Care and Use Committee and in accordance with the Chinese regulations and standards on the use of laboratory animals.

**Immunofluorescence assay**

To observe the expression of c-Myc in SLC34A2 reduced EJ and T24 cells, cells were cultured on 15mm diameter glass coverslips. After fixation in 4% paraformaldehyde and permeabilization using 0.3% Triton X-100, antibody against c-Myc (Abcam, Cambridge, UK) were incubated at 4°C overnight, followed by Alexa Fluor 594-conjugated secondary antibody (Life Technology) incubation. Nucleus was stained with 4’, 6-diamidino-2-phenylindole (DAPI, Sigma Aldrich). Slides were captured images with a confocal microscopy (Olympus, Japan).

**MiR-214 mimic, miR-214-mut, and reporter plasmids**

The 3'UTR of *SLC34A2* were amplified and cloned to a modified pGL3 control vector, downstream to the luciferase gene. MiR-214 mimic and miR-214-mut were purchased from RIBOBIO (Guangzhou, China). MiR-214 mimic, miR-214-mut, and 3'UTR of *SLC34A2* reporter plasmid were transfected into EJ and T24 cells using Lipofectamine2000 (Invitrogen), according to the manufacturer's instructions.

**Dual-luciferase reporter assay**

Cells were seeded into 24-well plates (4×10^4^/well) and 24 hours later transfected with a c-Myc or *SLC34A2* 3'UTR reporter plasmid (GeneCopoeia, Guangzhou, China). The renilla luciferase reporter pRL-TK was co-transfected as an internal control. The firefly and renilla luciferase activities were quantified 48 hours later using a Dual-Luciferase Reporter Kit (Promega) with a luminometer (Promega).
