# Supplementary material for: Overexpression of SLC34A2 is an independent prognostic indicator in bladder cancer and its depletion suppresses tumor growth via decreasing c-Myc expression and transcriptional activity
Source: Cell Death Dis. 2017 Feb 2;8(2):e2581–. doi: 10.1038/cddis.2017.13 (PMC5386463; doi:10.1038/cddis.2017.13)
Supplement: Supplementary Information [file cddis201713x2.doc]

**Supplementary Figure 1. Kaplan-Meier overall survival curves and log-rank test in BC patients stratified by Age (A and B), Gender (C and D), Grade (E and F), Tumor size (G and H), T statue (I and J), N statue (K and L), and Tumor multiplicity (M and N).**
