# Supplementary figures and images for: Overexpression of SLC34A2 is an independent prognostic indicator in bladder cancer and its depletion suppresses tumor growth via decreasing c-Myc expression and transcriptional activity
Source: Cell Death Dis. 2017 Feb 2;8(2):e2581–. doi: 10.1038/cddis.2017.13 (PMC5386463; doi:10.1038/cddis.2017.13)

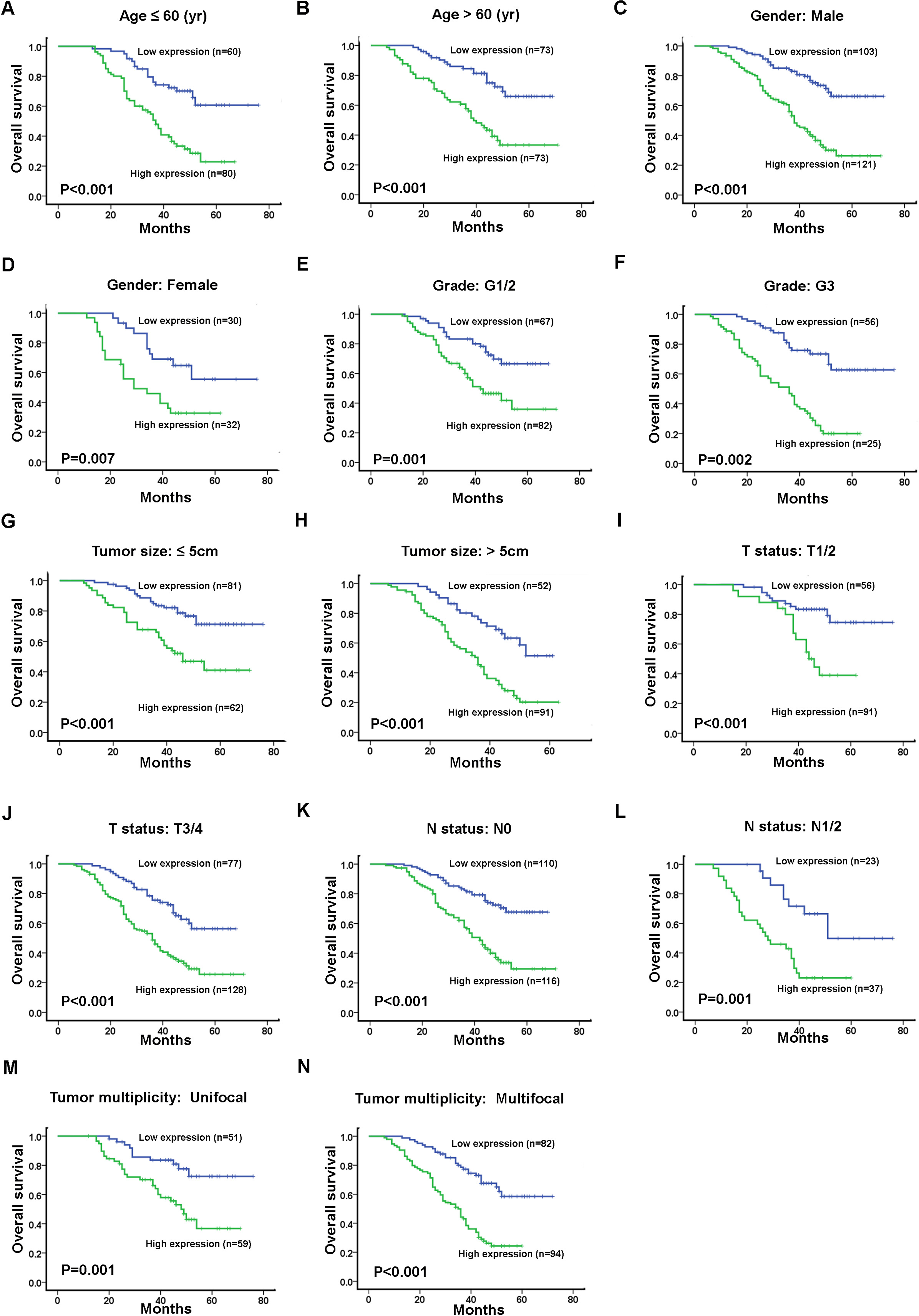

Supplement: Supplementary Information [file cddis201713x3.tif]
